# Repurposing Salicylanilide Anthelmintic Drugs to Combat Drug Resistant *Staphylococcus aureus*

**DOI:** 10.1371/journal.pone.0124595

**Published:** 2015-04-21

**Authors:** Rajmohan Rajamuthiah, Beth Burgwyn Fuchs, Annie L. Conery, Wooseong Kim, Elamparithi Jayamani, Bumsup Kwon, Frederick M. Ausubel, Eleftherios Mylonakis

**Affiliations:** 1 Division of Infectious Diseases, Rhode Island Hospital, Alpert Medical School of Brown University, Providence, Rhode Island, United States of America; 2 Division of Neurology, Rhode Island Hospital, Alpert Medical School of Brown University, Providence, Rhode Island, United States of America; 3 Massachusetts General Hospital, Harvard Medical School, Boston, Massachusetts, United States of America; Columbia University, UNITED STATES

## Abstract

*Staphylococcus aureus* is a Gram-positive bacterium that has become the leading cause of hospital acquired infections in the US. Repurposing Food and Drug Administration (FDA) approved drugs for antimicrobial therapy involves lower risks and costs compared to *de novo* development of novel antimicrobial agents. In this study, we examined the antimicrobial properties of two commercially available anthelmintic drugs. The FDA approved drug niclosamide and the veterinary drug oxyclozanide displayed strong *in vivo* and *in vitro* activity against methicillin resistant *S*. *aureus* (minimum inhibitory concentration (MIC): 0.125 and 0.5 μg/ml respectively; minimum effective concentration: ≤ 0.78 μg/ml for both drugs). The two drugs were also effective against another Gram-positive bacteria *Enterococcus faecium* (MIC 0.25 and 2 μg/ml respectively), but not against the Gram-negative species *Klebsiella pneumoniae*, *Acinetobacter baumannii*, *Pseudomonas aeruginosa* and *Enterobacter aerogenes*. The *in vitro* antimicrobial activity of niclosamide and oxyclozanide were determined against methicillin, vancomycin, linezolid or daptomycin resistant *S*. *aureus* clinical isolates, with MICs at 0.0625-0.5 and 0.125-2 μg/ml for niclosamide and oxyclozanide respectively. A time-kill study demonstrated that niclosamide is bacteriostatic, whereas oxyclozanide is bactericidal. Interestingly, oxyclozanide permeabilized the bacterial membrane but neither of the anthelmintic drugs exhibited demonstrable toxicity to sheep erythrocytes. Oxyclozanide was non-toxic to HepG2 human liver carcinoma cells within the range of its *in vitro* MICs but niclosamide displayed toxicity even at low concentrations. These data show that the salicylanilide anthelmintic drugs niclosamide and oxyclozanide are suitable candidates for mechanism of action studies and further clinical evaluation for treatment of staphylococcal infections.

## Introduction

*Staphylococcus aureus* has emerged as a primary cause of hospital and community-acquired infections [1, 2], often leading to bacteremia and sepsis [3, 4]. The virulence of this pathogen is manifested through its wide spectrum of virulence factors and strategies to bypass host defense mechanisms [5, 6]. Unfortunately, the rising tide of antibiotic resistance, especially against β-lactam antibiotics, such as methicillin, has severely constrained treatment options. In 2011, the Centers for Disease Control and Prevention (CDC) estimated that methicillin resistant *S*. *aureus* (MRSA) was associated with 80,461 life-threatening infections, resulting in 11,285 fatalities in the US alone and that the yearly mortality rate surpassed that of Acquired immunodeficiency syndrome (AIDS) [7, 8]. Therefore, novel anti-infectives and treatment strategies are urgently needed against *S*. *aureus*.

Classical antimicrobial drug discovery involves *in vitro* screening for antimicrobial candidates, Structure Activity Relationship (SAR) analysis, followed by *in vivo* testing for toxicity [9]. Bringing drugs from the bench to the bedside involves huge expenditures in time and resources. This, along with the relatively short window of therapeutic application for antibiotics attributed to the rapid emergence of drug resistance, has, at least until recently, resulted in a waning interest in antibiotic discovery among pharmaceutical companies [10]. In this environment, “repurposing” (defined as investigating new uses for existing drugs) has gained renewed interest, as reflected by several recent studies [11–13].

The free living nematode *C*. *elegans* has recently become a popular model organism for studying pathogenesis of many microbes [14–17], including *S*. *aureus* [18, 19]. Using *C*. *elegans* as a platform for antimicrobial drug discovery enables simultaneous assessment of toxicity and efficacy of the test compounds, which lowers the burden for further animal testing [20]. We have developed a high throughput *C*. *elegans*-MRSA liquid infection assay and screened a compound library consisting of bioactives and FDA approved drugs for compounds that prolong survival of *C*. *elegans* infected with MRSA [21]. An unexpected finding from that study was that, although a nematode was used as a model host, the anthelmintic drug closantel was identified as a hit that prolonged survival of the infected worms. Moreover, the *in vivo* and *in vitro* activity of closantel was comparable to that of vancomycin, a drug of last resort that is widely used to combat MRSA infections [22].

The identification of closantel as a potent anti-staphylococcal drug [21, 23] motivated us to determine the anti-staphylococcal properties of niclosamide and oxyclozanide, two analogs of closantel. All three compounds belong to the salicylanilide family of anthelmintic drugs. Niclosamide is an FDA approved drug widely used for treating tape worm infection in humans and has lately been shown to possess anti-cancer and anti-diabetic activities [24–26]. Niclosamide is also listed in the World Health Organization (WHO) model list of essential medicines [27]. Oxyclozanide is used in veterinary medicine, primarily for ruminants such as cattle, goat and sheep to control parasitic flatworms called flukes [28, 29]. The antimicrobial properties of salicylanilide derivatives have recently been described [30–33]. Cheng et al. [30] used a high-throughput screening strategy to identify a salicylanilide derivative that inhibits MRSA cell wall synthesis and Kratky et al. [31, 33] and Pauk et al. [32] described the synthesis of salicylanilide analogues that exhibited antimicrobial activity against a variety of bacteria including MRSA and mycobacteria. However, the studies mentioned above did not involve salicylanilide drugs that are currently in use, such as niclosamide and oxyclozanide.

We report here that niclosamide and oxyclozanide were effective against MRSA both *in vitro* and *in vivo* in the *C*. *elegans* infection model, and that the mode of action of oxyclozanide may involve bacterial membrane disruption. Niclosamide displayed toxicity to mammalian cells even at low concentrations whereas oxyclozanide displayed toxicity only at concentrations higher than the effective concentration against MRSA. The findings from this study suggest the advisability of further studies on the antibacterial mechanism of action of salicylanilide anthelmintic drugs and their clinical potential for treating staphylococcal infections.

## Materials and Methods

### Bacterial and nematode strains

All bacterial strains tested in this study were selected from an existing strain collection maintained in our laboratory. The following strains (Tables 1 and 2) were used to evaluate the antibacterial activities of anthelmintic drugs: *S*. *aureus* (MRSA MW2, Newman, RN4220, RN6390, USA100, USA300, USA400), *S*. *epidermidis* (9142), *Enterococcus faecium* (E007), *Klebsiella pneumoniae* (ATCC 77326), *Acinetobacter baumannii* (ATCC 17978), *Pseudomonas aeruginosa* (PA14), *Enterobacter aerogenes* (EAE 2625) and 44 *S*. *aureus* clinical isolates. All bacterial strains were grown at 37°C. Staphylococcal strains were grown in tryptic soy broth (TSB); *K*. *pneumoniae*, *A*. *baumannii*, *P*. *aeruginosa* and *E*. *aerogenes* were grown in Luria-Bertani broth (LB) and *E*. *faecium* was grown in Brain-heart infusion medium (Becton Dickinson and Company, Franklin Lakes, NJ, USA).

**Table 1:** Comparative antibacterial activities of niclosamide, oxyclozanide, vancomycin and polymyxin B against ESKAPE pathogens.

Strain	Niclosamide (μg/ml)		Oxyclozanide (μg/ml)		Vancomycin (μg/ml)		Polymyxin B (μg/ml)	
	MIC	MBC	MIC	MBC	MIC	MBC	MIC	MBC
*E*. *faecium* (E007) [65]	0.25	>64	2	32	4	64	>64	ND
*S*. *aureus* (MW2) [66]	0.125	32	0.5	2	4	16	64	>64
*K*. *pneumoniae* (ATCC 77326)	>64	ND	>64	ND	>64	ND	8	8
*A*. *baumannii* (ATCC 17978)	>64	ND	>64	ND	>64	ND	2	4
*P*. *aeruginosa* (PA14) [67]	>64	ND	>64	ND	>64	ND	1	2
*E*. *aerogenes* (EAE 2625)	>64	ND	>64	ND	>64	ND	8	8

**Table 2:** Antibacterial activities of niclosamide and oxyclozanide against staphylococcal strains.

Strain	Niclosamide (μg/ml)	Oxyclozanide (μg/ml)	Oxacillin (μg/ml)	Vancomycin (μg/ml)	Daptomycin (μg/ml)	Telavancin (μg/ml)
MW2 [66]	0.125	0.5	64	4	8	1
Newman [68]	< = 0.0625	< = 0.0625	< = 0.0625	2	4	0.5
RN4220 [69]	< = 0.0625	< = 0.0625	< = 0.0625	2	4	0.125
RN6390 [70]	< = 0.0625	< = 0.0625	< = 0.0625	2	4	< = 0.0625
USA100 [71]	< = 0.0625	< = 0.0625	< = 0.0625	2	4	0.5
USA300 [71]	< = 0.0625	< = 0.0625	>64	4	8	1
USA400 [71]	< = 0.0625	< = 0.0625	>64	2	4	1
9142^*^ [72]	0.5	0.125	>64	4	2	< = 0.0625

* is an *S*. *epidermidis strain*, rest are *S*. *aureus* strains.

The *C*. *elegans glp-4(bn2);sek-1(km4)* double mutant strain were maintained at 15°C on a lawn of *E*. *coli* strain HB101 on 10 cm plates as described [34]. The *glp-4(bn2)* mutation renders the strain incapable of producing progeny at 25°C [35] and the *sek-1(km4)* mutation enhances sensitivity to various pathogens [36], reducing assay time.

### *C*. *elegans*-MRSA liquid infection assay

The *C*. *elegans*-MRSA liquid infection assay has been described [21, 37]. In brief, 4,500 *glp-4(bn2);sek-1(km4)* L1 hatchlings were grown on SK-HB101 agar plates for 52 hours at the restrictive temperature of 25°C until animals became sterile young adults and later harvested with M9 buffer. *S*. *aureus* MW2 was grown overnight at 37°C in TSB, first under aerobic conditions and later shifted to anaerobic growth conditions at 37°C. The infection assay was performed in standard 384-well assay plates (Corning no. 3712; Corning, Corning, NY, USA), in the presence of the compound being tested or 1% dimethyl sulfoxide (DMSO) as control. Bacteria were added to the wells at a final OD_600_ of 0.04, followed by the use of a Complex Object Parametric Analyzer and Sorter (COPAS) large particle sorter (Union Biometrica, Holliston, MA, USA) to transfer 15 adult worms to each well of an assay plate. After 5 days of incubation in a humidified chamber at 25°C, the bacteria and other debris were washed from the wells with a microplate washer and the worms were stained with the vital dye Sytox Orange (Life Technologies, Carlsbad, CA, USA). After overnight incubation at 25°C in a humidified chamber, the plates were imaged using an Image Xpress Micro automated microscope (Molecular Devices, Sunnyvale, CA, USA), capturing both transmitted light and TRITC (535 nm excitation, 610 nm emission) fluorescent images with a 2X objective.

The images from the infection assay were processed using the open source image analysis software CellProfiler (http://www.cellprofiler.org/) and analysis modules as described previously [38, 39]. The ratio of Sytox worm area to bright field worm area, and the resultant percentage survival data, is calculated by the software for each well of the assay plates. The entire assay was performed in duplicate.

### Antimicrobial susceptibility testing

Compounds (5 mg/ml stock solution in DMSO) were tested by broth microdilution and disc diffusion assays in triplicate, adapted from established protocols [40]. Broth microdilution was performed in triplicate in 96-well plates using Müller-Hinton broth (Becton Dickinson and Company, Franklin Lakes, NJ, USA). For testing MIC with daptomycin, the media was supplemented with CaCl_2_ at a final concentration of 50 μg/ml. The assay volume was 100 μl and two-fold serial dilutions were carried out to get compounds in the concentration range 0.0625–64 μg/ml. The bacterial concentration was adjusted to an initial OD_600_ of 0.03. After overnight incubation at 35°C, the absorbance was measured to determine antimicrobial activity. The minimum bactericidal concentration (MBC) was determined by plating 10 μl of culture volume from the MIC assay on Müller-Hinton agar and colony formation was examined after overnight incubation at 35°C. The lowest concentration at which colonies were not observed was regarded as the MBC.

The disc diffusion test was carried out in triplicate on Müller-Hinton agar (Becton Dickinson and Company, Franklin Lakes, NJ, USA). Discs made from Whatman filter paper (GE Healthcare, Little Chalfont, Buckinghamshire, United Kingdom) using a paper punch were impregnated with 25 μg of test compounds and air-dried. Three hundred microliters of an overnight bacterial culture was spread on an agar plate and air-dried. The antimicrobial discs were overlaid on the plate and incubated at 35°C for 20 hrs. Antimicrobial susceptibly was determined by measuring the diameter of the zone of inhibition.

### Time-kill study

The bactericidal properties of test compounds were assessed using a time-kill study, adapted from a protocol described earlier [41]. The assay was conducted in 5 ml round bottomed tubes (BD Biosciences no. 352235; BD Biosciences, San Jose, CA, USA). An overnight culture of MRSA (MW2) was diluted in fresh TSB to a density of 10^6^ cells/ml. Test compounds, at 4x their MIC concentration, were added to each tube and incubated with shaking at 37°C. At periodic intervals, aliquots sampled from the tubes were serially diluted and plated on tryptic soy agar (TSA; Becton Dickinson and Company, Franklin Lakes, NJ, USA) plates. The plates were incubated overnight at 37°C and the colonies were counted to measure viability. The assay was done in duplicate.

### Bacterial cell membrane permeabilization assay

Permeabilization of bacterial membranes was determined by Sytox Green (Life Technologies, Carlsbad, CA, USA) uptake by bacterial cells in 96 well plates (Corning CLS3300; Corning, Corning, NY, USA) as described in an earlier study [42]. The assay was done in triplicate. MRSA strain MW2 cells in logarithmic growth phase were harvested by centrifugation at 4000 rpm for 5 minutes, the pellet was washed twice in phosphate-buffered saline (PBS), and resuspended in PBS to an absorbance of 0.5 at 595 nm. Sytox Green was added to the cells at a final concentration of 5 μM and incubated in the dark for 30 minutes. Fifty microliters of cell suspension was added to 50 μl of compounds serially diluted in PBS. The fluorescence intensities were measured at different time points, with the excitation and emission at 485 nm and 530 nm respectively. The assay was repeated twice.

### Bacterial lysis assay

The assay for testing the ability of the antimicrobial compounds to induce lysis of bacterial cells was adapted from a protocol described in [43]. Briefly, logarithmically growing MRSA (MW2) cells were harvested by centrifugation at 4000 rpm for 5 minutes, washed twice with PBS, and resuspended in PBS to an absorbance of 0.5 at 595 nm. The assay was carried out in triplicate in a 96 well plate by adding 50 μl of cell suspension to 50 μl of compounds serially diluted in PBS, and incubating at 37°C. The absorbance at 595 nm was measured at periodic time points and the relative absorbance was calculated by dividing the absorbance of each well with that of the negative control (DMSO). All assays were performed in triplicate.

### Sheep blood hemolysis

The protocol to test the ability of compounds to cause hemolysis of sheep erythrocytes (Rockland Immunochemicals, Limerick, PA, USA) was adapted from Rosch et al. [44]. In a 96 well plate, 50 μl of 2% sheep erythrocytes suspended in PBS was added to 50 μl of compounds serially diluted in PBS and incubated at 37°C for 1 hour. The plate was then centrifuged at 500 G for 5 minutes and 50 μl of the supernatant from each well of the assay plate was transferred to a fresh 96 well plate. Hemolysis was confirmed by both visual observation and measuring absorbance at 540 nm. Treatment was conducted in triplicates.

### Cytotoxicity assay

The protocol for measuring cytotoxicity was described in Kwon et al. [45]. HepG2 cells were cultured in Dulbecco’s modified Eagle medium (DMEM; Life Technologies, Carlsbad, CA, USA) containing 10% fetal bovine serum, 25 mM D-glucose, 2 mM L-glutamine, 1 mM sodium pyruvate and 1% penicillin/streptomycin and maintained at 37°C in 5% CO_2_. For the toxicity test, HepG2 (ATCC HB 8065; ATCC, Manassas, VA, USA) were cultured at 70–80% confluence in 96-well plates in a volume of 100 μl/well culture medium. Serially diluted chemical compounds were incubated with the cells at 37°C in 5% CO_2_ for 24 hours. Ten microliters of 2-(4-iodophenyl)-3-(4-nitrophenyl)-5-(2, 4-disulfophenyl)-2*H*-tetrazolium (WST-1) solution (Roche, Mannheim, Germany) were added per well for the last 4 hours of the 24 hours period. WST-1 reduction was detected using absorbance at 490 nm by a Vmax microplate reader (Molecular Device, Sunnyvale, CA, USA). The percent fluorescence relative to that of the no-treatment control was calculated. The assay was done in triplicate.

## Results

### Salicylanilide anthelmintic drugs prolong worm survival in the *C*. *elegans*-MRSA liquid infection assay

**Fig 1:**
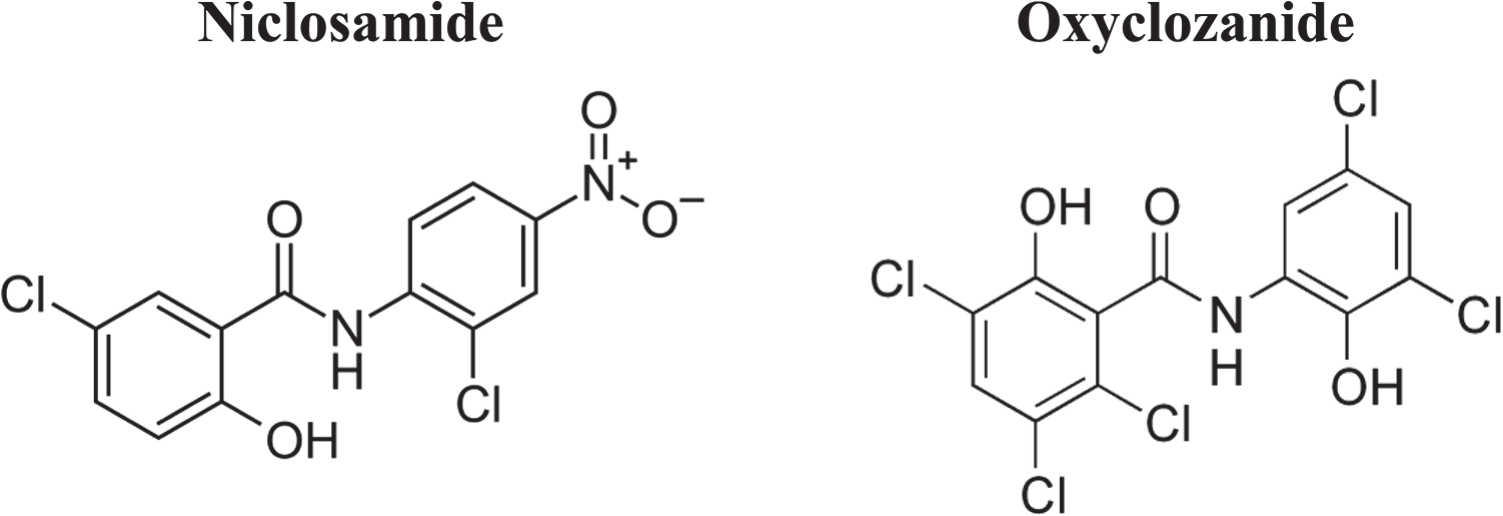
Chemical structure of the salicylanilide anthelmintic drugs niclosamide and oxyclozanide.

**Fig 2:**
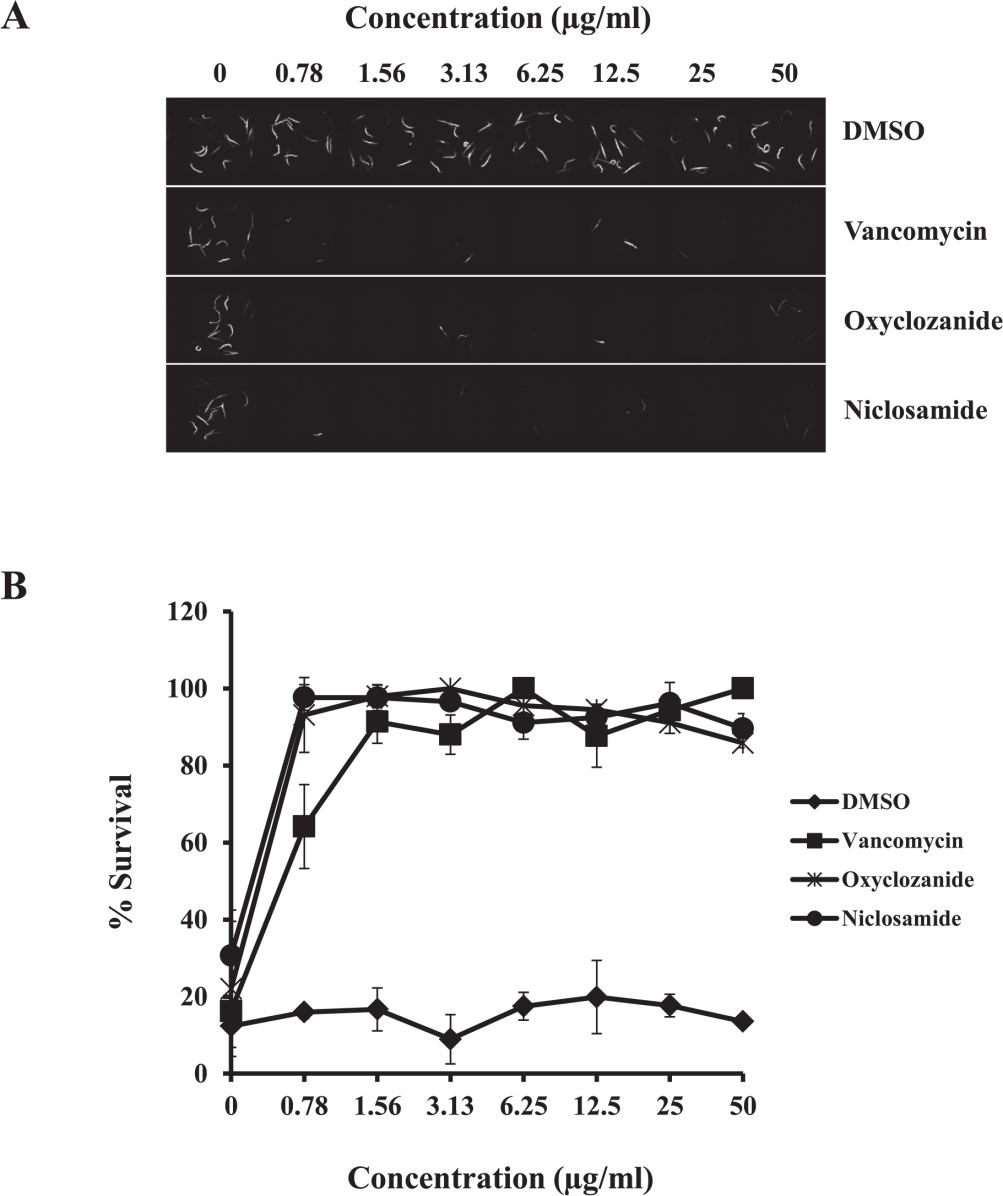
Salicylanilide anthelmintic drugs prolong survival of *C*. *elegans* infected with MRSA. A 384 well assay plate was co-inoculated with nematodes, bacteria and either DMSO (negative control), vancomycin (positive control) or the salicylanilide anthelmintic drugs. Serial dilutions of the drugs were tested to adjust the concentration between 0.78–50 μg/ml. **(A)** Sytox Orange stained and bright field images of assay wells containing a gradient of the tested drugs. Dead worms take up the vital dye Sytox Orange and fluoresce. **(B)** Percent survival of infected worms was calculated from intensity of fluorescence measured in each well of the assay plate.

In a previous study, we established a *C*. *elegans*-MRSA whole animal liquid infection assay for high throughput screening of chemical libraries to identify anti-infectives that prolong survival of infected worms [21]. The Biomol 4 library comprising of 640 FDA-approved drugs was screened and one of the hits identified was closantel, a salicylanilide veterinary anthelmintic drug used to control nematode and trematode infection of the intestines and liver [46]. The Biomol 4 library also included 13 other non-salicylanilide anthelmintic drugs (S1 Fig), none of which prolonged survival of *C*. *elegans* infected with MRSA. Closantel demonstrated good i*n vitro* and *in vivo* activity against MRSA at a low concentration of 0.78 μg/ml and was only mildly toxic to *C*. *elegans* even at a high concentrations (>50 μg/ml). These findings motivated us to investigate other commercially available salicylanilide anthelmintic drugs, such as niclosamide and oxyclozanide (Fig 1) for potential antimicrobial properties. The *in vivo* antimicrobial activity of niclosamide and oxyclozanide were compared with vancomycin in the *C*. *elegans*-MRSA liquid infection assay. Niclosamide and oxyclozanide behaved similarly to vancomycin in prolonging survival of infected worms at the lowest tested concentration of 0.78 μg/ml (Fig 2).

### Salicylanilide anthelmintic drugs inhibit Gram-positive bacteria in the ESKAPE panel

**Fig 3:**
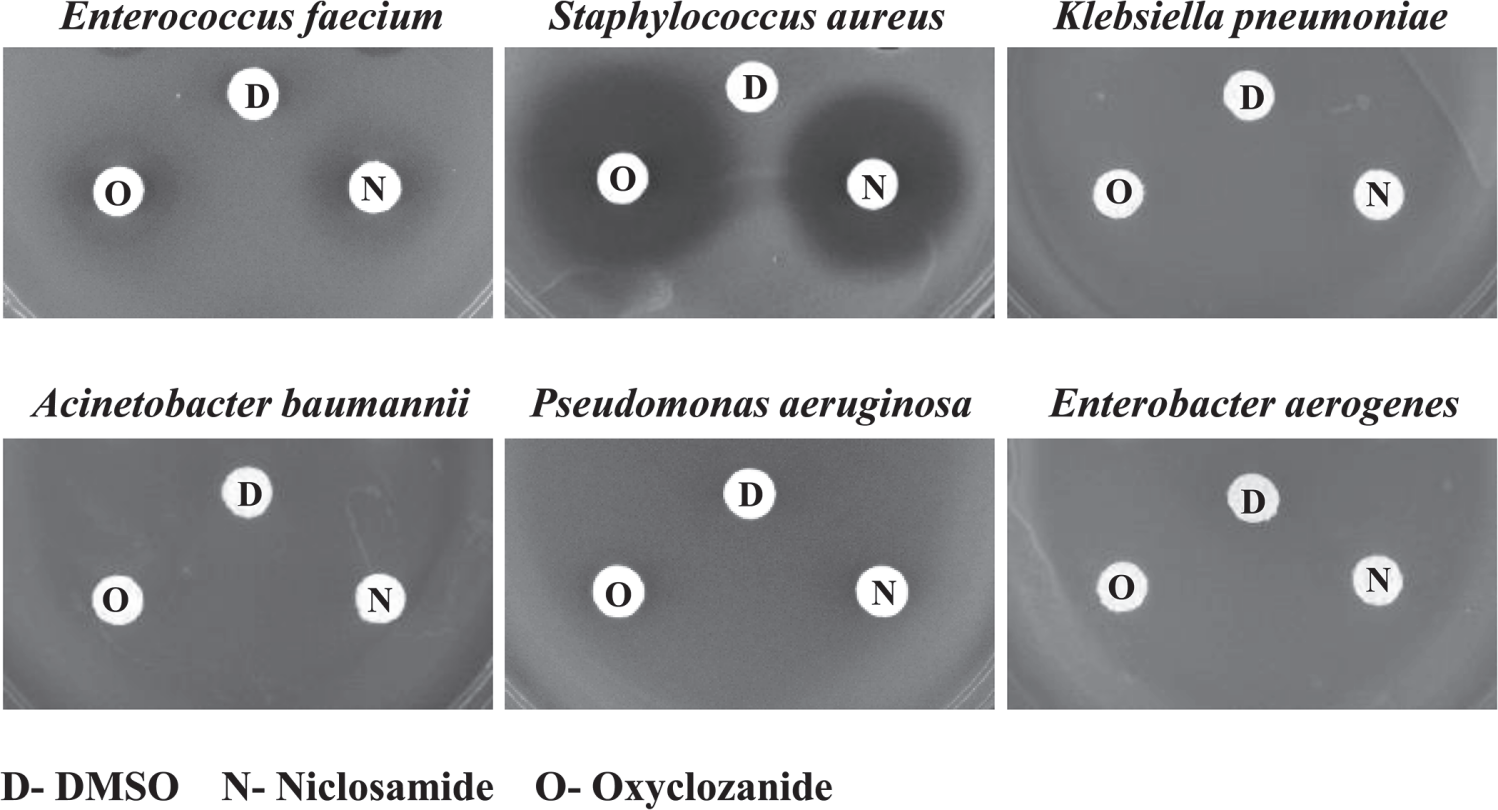
Antimicrobial activity of niclosamide and oxyclozanide against the ESKAPE pathogens. Disc diffusion assay to detect zone of growth inhibition due to bactericidal activity of niclosamide and oxyclozanide against *E*. *faecium*, *S*. *aureus* methicillin resistant strain (MW2), *K*. *pneumoniae*, *A*. *baumannii*, *P*. *aeruginosa* and *E*. *aerogenes*.

The ESKAPE pathogens *E*. *faecium*, *S*. *aureus*, *K*. *pneumoniae*, *A*. *baumannii*, *P*. *aeruginosa* and *Enterobacter* spp. are the main causes of nosocomial infections, which are often difficult to treat due to development of antimicrobial resistance [47]. The *in vitro* antimicrobial activity of niclosamide and oxyclozanide were evaluated using agar disc diffusion and broth microdilution assays (Fig 3 and Table 1). In the case of the *S*. *aureus* (MRSA strain MW2), niclosamide and oxyclozanide formed large clear zones of growth inhibition and the MICs of niclosamide and oxyclozanide were 0.125 and 0.5 μg/ml respectively and the MBCs for the two drugs were 32 and 2 μg/ml respectively (S2B Fig). For *E*. *faecium*, growth inhibition zones for niclosamide and oxyclozanide appeared somewhat diffused. The MICs of niclosamide and oxyclozanide against *E*. *faecium* were 0.25 and 2 μg/ml respectively and the MBCs of the same drugs were >64 and 32 μg/ml respectively (S2A Fig). The 2 salicylanilide anthelmintic drugs displayed no zones of growth inhibition against *K*. *pneumoniae*, *A*. *baumannii*, *P*. *aeruginosa* and *E*. *aerogenes* and did not inhibit bacterial growth in the broth microdilution assays even at the maximum tested concentration of 64 μg/ml.

### Niclosamide is bacteriostatic and oxyclozanide is bactericidal against *S*. *aureus*

**Fig 4:**
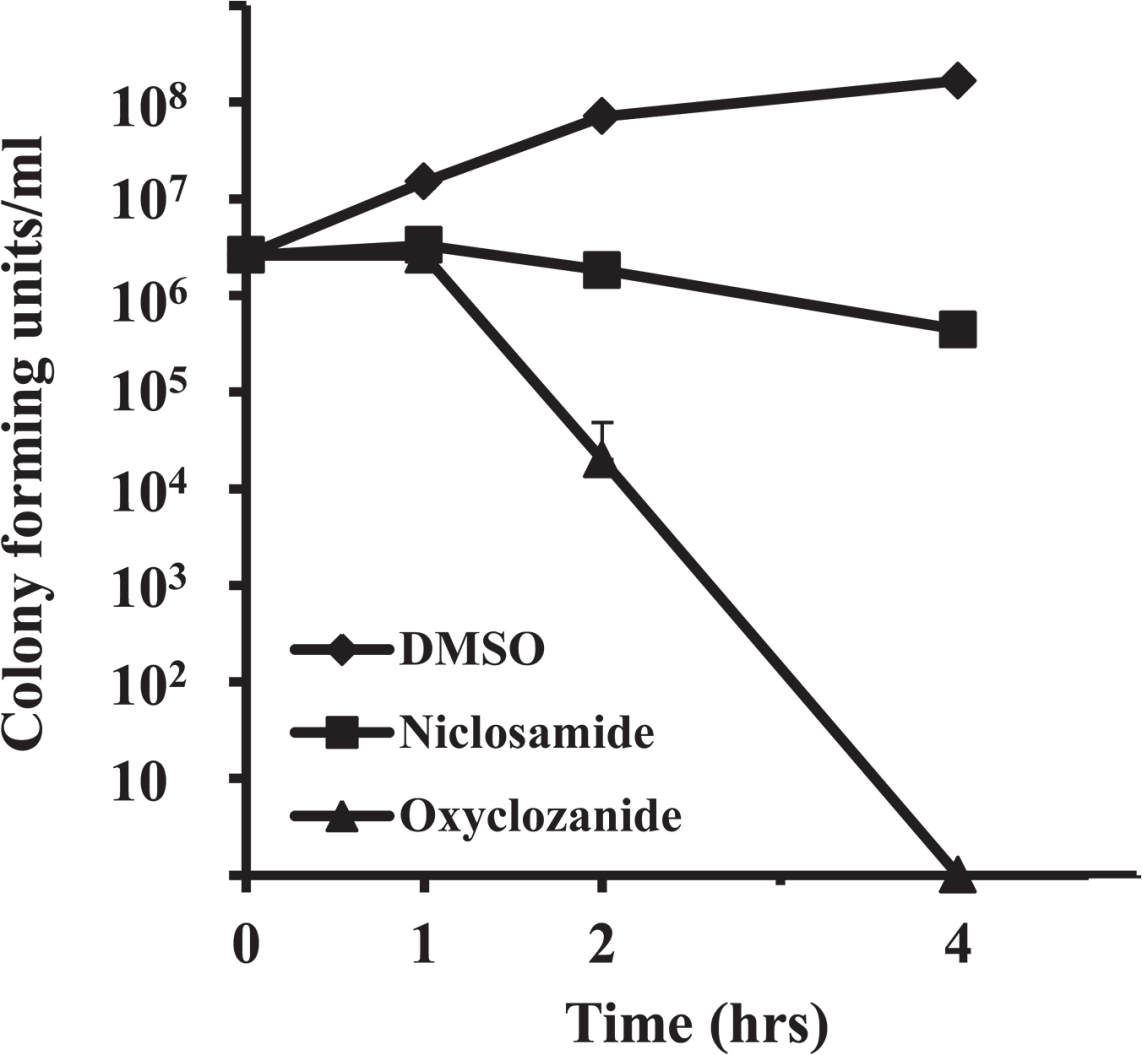
Comparative time-kill kinetics of niclosamide and oxyclozanide against MRSA. The survival of MW2 in a broth culture treated with DMSO, niclosamide or oxyclozanide at a concentration of 4 times the MIC. (MIC values: niclosamide 0.125 μg/ml, oxyclozanide 0.5 μg/ml).

An antibacterial compound is generally regarded as bactericidal if the MBC is no more than four times the MIC and it is regarded as bacteriostatic for MBC levels beyond this range [48]. Based on the data presented in the previous section, the MBC/MIC ratios of niclosamide, and oxyclozanide against *S*. *aureus* (MRSA-MW2) were 256 and 4 respectively suggesting that niclosamide is bacteriostatic, whereas oxyclozanide is bactericidal. To further explore the nature of action against MRSA-MW2, a time-kill assay was performed with niclosamide and oxyclozanide at a concentration of 4xMIC. Oxyclozanide was able to kill MRSA completely during a 4 hour exposure period. During the same exposure period, niclosamide only caused a six fold drop in colony forming units (CFU) (Fig 4), showing that niclosamide is bacteriostatic whereas oxyclozanide is bactericidal against *S*. *aureus*.

### Oxyclozanide disrupts the *S*. *aureus* cell envelope but does not induce cell lysis

**Fig 5:**
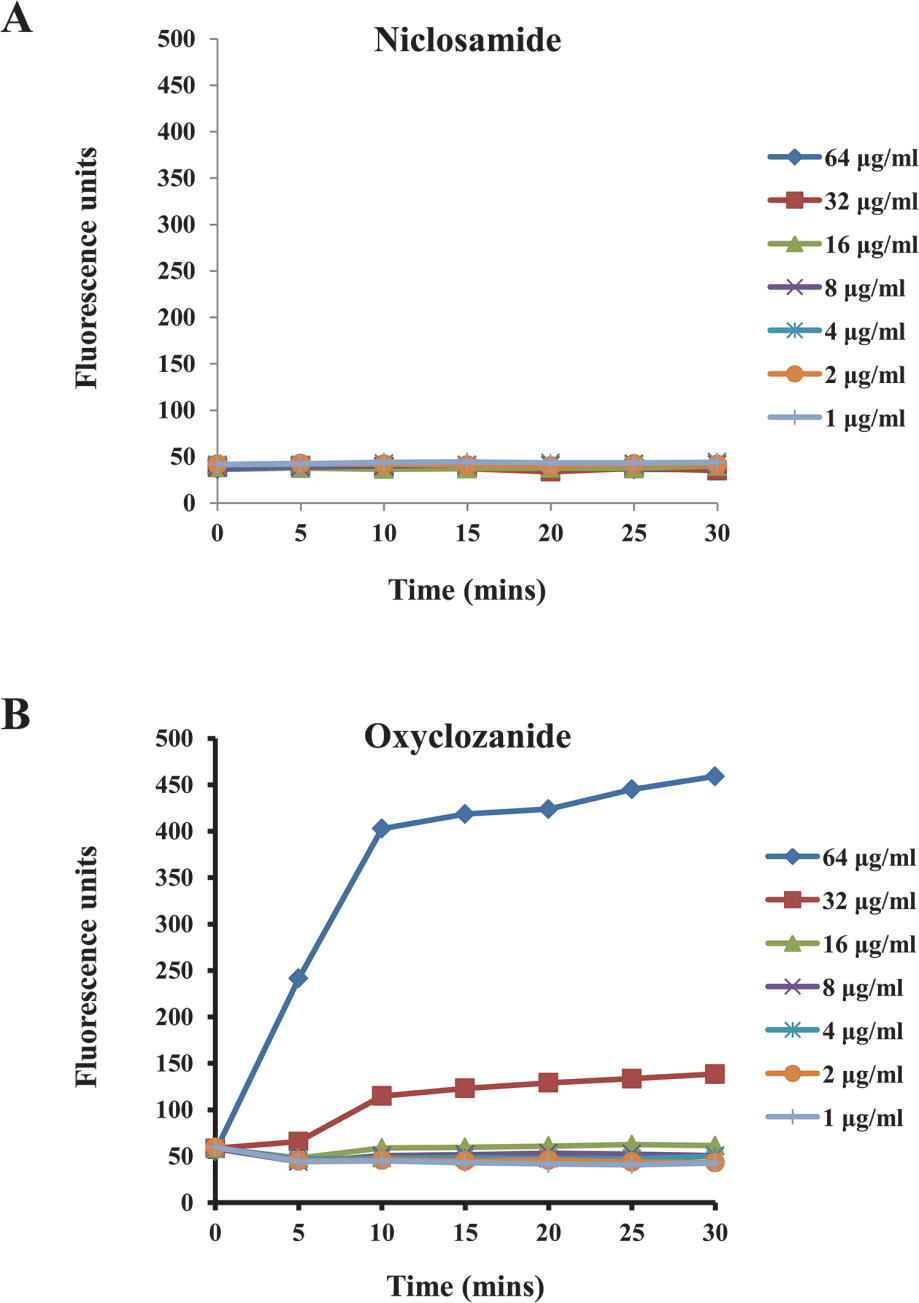
Permeabilization of MRSA cells by niclosamide and oxyclozanide. Time course of membrane permeabilization by oxyclozanide **(A)** and niclosamide **(B)** was measured by following the increase in Sytox Green fluorescence (λexc = 485 nm; λem = 530 nm). The drugs were tested at serially diluted concentrations between 1 to 64 μg/ml.

In order to investigate the antibacterial mode of action of the 2 salicylanilide anthelmintic drugs, the potential of these drugs to cause disruption of the bacterial cell envelope was examined. MRSA-MW2 cells were exposed to niclosamide or oxyclozanide at concentrations between 1–64 μg/ml and bacterial membrane disruption was evaluated by studying uptake of the DNA staining dye Sytox Green over a period of 30 minutes. In cells treated with oxyclozanide, a dose dependent uptake of Sytox Green was observed as represented by the increase in cellular fluorescence caused by binding of the dye with the bacterial DNA (Fig 5B). However, cells treated with niclosamide showed no uptake of the dye even at a high concentration of 64 μg/ml (Fig 5A), suggesting that the cell envelope remained intact.

To determine if oxyclozanide also induces lysis of MRSA cells apart from causing membrane damage, a bacterial lysis assay was performed according to the protocol detailed in the Methods section. Niclosamide and oxyclozanide were tested at concentrations between 1–64 μg/ml. Cells treated with lysostaphin, a known membrane disrupting compound that causes cell lysis, at the same concentration range were used as positive control, and DMSO treated cells were the negative control. Lysostaphin caused near complete lysis of bacterial cells as early as 15 minutes after the start of the assay as reflected by the relative absorbance dropping nearly to 0. However, the anthelmintic drugs did not cause any reduction of the absorbance of MRSA even after 8 hours of incubation at the maximum tested concentration of 64 μg/ml (data not shown), suggesting that oxyclozanide disrupts the bacterial cell envelope without causing lysis of the cell.

### Niclosamide and oxyclozanide do not cause hemolysis

**Fig 6:**
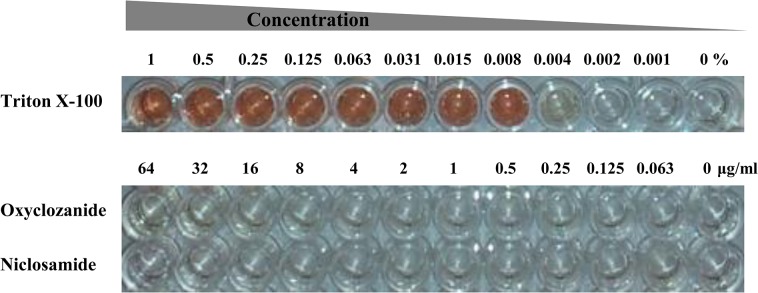
Hemolytic activity of niclosamide and oxyclozanide. Sheep erythrocytes were treated with serial dilutions of Triton X-100 (.001–1%), oxyclozanide and niclosamide (0.063–64 μg/ml)

The ability of oxyclozanide to cause disruption of the bacterial cell envelope raises the possibility of a similar activity against mammalian cell membranes. To test this possibility, the ability of niclosamide and oxyclozanide to induce hemolysis of sheep erythrocytes was examined. Sheep red blood cells (RBCs) were treated with serial dilutions of the anthelmintic drugs (0.063–64 μg/ml) for 1 hour. Cells treated with serial dilutions of triton X-100 (0.001–1% solution) were the positive control. Neither of the anthelmintic drugs caused any RBC hemolysis even at the maximum tested concentration of 64 μg/ml (Fig 6), which was also confirmed by measuring absorbance at 540 nm (data not shown). The positive control triton X-100 caused hemolysis at all concentrations starting from 0.008%.

### Cytotoxicity of niclosamide and oxyclozanide against HepG2 human liver carcinoma cells

**Fig 7:**
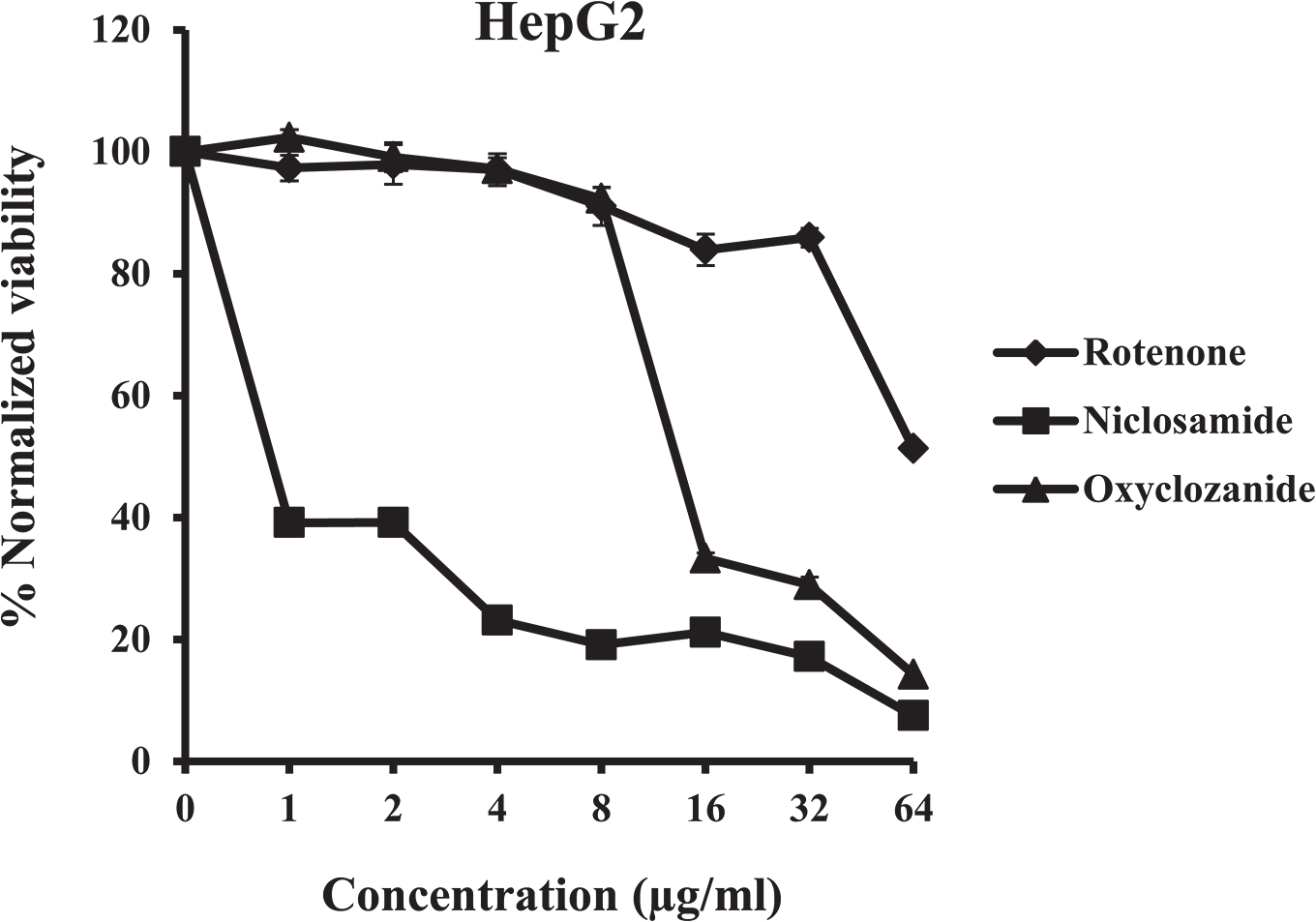
Cytotoxicity of niclosamide and oxyclozanide to HepG2. The viability of HepG2 cells was measured after treatment with serially diluted concentrations (1–64 μg/ml) of niclosamide, oxyclozanide or the mitochondrial toxin rotenone (positive control). Cell viability was measured spectrophotometrically by detecting degradation of WST-1 dye into formazan by viable cells, which produces an intense color.

Niclosamide is an FDA approved drug and has been in clinical use for several decades. In the case of oxyclozanide, though the drug has been extensively tested in animals, no information is available on effects in humans. The cytotoxicity of niclosamide and oxyclozanide was evaluated against HepG2, a cell line derived from human liver carcinoma cells. HepG2 cells were treated with serial dilutions of the anthelmintic drug between the concentration range 1–64 μg/ml and cellular viability was measured. The mitochondrial toxin rotenone, which interferes with electron transport chain in mitochondria, was used as the positive control. Cells treated with oxyclozanide were almost 100% viable for drug concentrations up to 8 μg/ml and viability dropped with further increase in concentration. Niclosamide displayed toxicity, 40% of the drug treated cells were viable at the lowest tested concentration of 1 μg/ml and viability dropped even further with increasing drug concentrations (Fig 7).

### Salicylanilide anthelmintic drugs are effective against drug resistant *S*. *aureus* clinical isolates and against staphylococci of different genetic backgrounds

The antimicrobial activity of niclosamide and oxyclozanide was evaluated against the following drug-resistant clinical staphylococcal isolates: 26 MRSA isolates (methicillin MICs ≥ 16 μg/ml and vancomycin MICs between 1–2 μg/ml), 3 vancomycin resistant isolates (vancomycin MICs ≥ 8 μg/ml), 4 linezolid resistant isolates (linezolid MICs ≥8 μg/ml) and 11 daptomycin resistant isolates (daptomycin MICs ≥4 μg/ml). Niclosamide and oxyclozanide displayed MICs between 0.0625–0.5 and 0.5–2 μg/ml respectively against all tested clinical isolates (data not shown). The antimicrobial activity of niclosamide and oxyclozanide was also evaluated against staphylococci of diverse genetic backgrounds and their *in vitro* activity was compared with commercially available anti-staphylococcal drugs such as telavancin, oxacillin, vancomycin and daptomycin. Both niclosamide and oxyclozanide were as effective as the commercial anti-staphylococcal drugs and displayed MICs between 0.0625–0.5 μg/ml for both drugs (Table 2).

## Discussion and Conclusions

The aim of this study was to investigate the antimicrobial properties of niclosamide and oxyclozanide, which are anthelmintic drugs belonging to the salicylanilide structural family [49]. Niclosamide is primarily used for treating tape worm infection in humans and oxyclozanide is a veterinary drug used for treating fluke infection in ruminants. To our knowledge this is the first study to systematically study the antimicrobial properties of salicylanilide anthelmintic drugs against nosocomial pathogens, including clinical isolates, and also test them in an *in vivo* infection model. The 2 anthelmintic drugs act specifically against Gram-positive bacteria and were as effective as vancomycin in inhibiting MRSA both *in vitro* and in the *C*. *elegans* whole animal model. Though the mode of action of niclosamide against MRSA is unclear, the antimicrobial activity of oxyclozanide potentially involves causing disruption to the bacterial cell envelope. Neither of the anthelmintic drugs was hemolytic against sheep RBCs. Niclosamide and oxyclozanide were further tested for toxicity against mammalian cells. Oxyclozanide was found to be non-toxic within the range of the drug MICs whereas niclosamide displayed some toxicity even at the lowest tested concentration of 1 μg/ml.

Earlier studies have documented the antimicrobial properties of the anthelmintic drug closantel [21, 23]. Macielag et al. demonstrated that closantel inhibits the KinA/Spo0F two-component regulatory system in *Bacillus subtilis*. In the same study the authors determined the MIC of closantel against *S*. *aureus*, *E*. *faecalis* and *E*. *faecium* to be in the range 0.12–1 μg/ml. The *in vivo* antimicrobial activity of closantel was also shown by our group in a previous study where we established a *C*. *elegans*-MRSA liquid infection assay for high throughput screening of small molecule libraries [21]. In both studies mentioned above, the MIC of closantel was well below that of vancomycin, attesting to the potential importance of this class of anthelmintic drugs as anti-staphylococcal agents, especially in light of the paucity of drugs with novel structural features for treating Gram-positive bacterial infections.

Salicylanilide anthelmintic drugs are in most circumstances consumed orally. There is very little absorption or metabolism of these drugs in the intestine and the systemic circulation of these drugs and their metabolites is also minimal [50, 51]. Since oxyclozanide is used in veterinary medicine, earlier studies have focused mostly on the drug’s pharmacology in ruminants [50]. Niclosamide on the other hand has been used in humans for several decades and detailed studies on the pharmacological profile of this drug are available [51]. In both humans and animals, salicylanilide anthelmintic drugs are extensively plasma bound which would explain the poor distribution in tissues. Human volunteers given an oral dose of 2,000 mg niclosamide eliminated the drug within 2 days. The fraction eliminated in urine was up to 25% and the rest was eliminated with feces. It was also found that the maximal serum concentration of niclosamide was 0.25–6 μg/ml, which is within the concentration range at which niclosamide inhibited growth of *S*. *aureus* as determined from the MIC assay (Table 2). However, the low level of systemic circulation coupled with the rapid elimination profile of niclosamide suggests the necessity for further testing of the potential of niclosamide and oxyclozanide for treating systemic infections. Further studies should include the evaluation of these compounds in systemic and localized infection models in rodents and the evaluation of derivative compounds.

Both anthelmintic drugs behaved similarly to vancomycin in prolonging survival of *C*. *elegans* infected with MRSA (Fig 2). The *C*. *elegans*-*S*. *aureus* infection model has been widely used to study staphylococcal virulence and pathogenesis [18, 19, 52–55]. Key virulence factors that are important for staphylococcal pathogenesis in the nematode model are also involved in pathogenesis in humans [18]. Apart from studying pathogen virulence and host response, the nematode infection model can also be used for high throughput screening of chemical libraries, as demonstrated by recent studies carried out with *P*. *aeruginosa* and *S*. *aureus* [21, 56]. One of the important advantages in the *C*. *elegans* infection model is that while testing the antimicrobial properties of a compound, toxicity can be assessed simultaneously since toxic compounds will kill the nematodes [57]. Although it may appear surprising that the anthelmintic drugs being tested were non-toxic to *C*. *elegans*, a recent study which compared the effect of different classes of anthelmintic drugs, such as albendazole, ivermectin, pyrantel and nitazoxanide (analogue of niclosamide) on diverse nematodes, reported that only pyrantel was slightly toxic to *C*. *elegans* [58].

The anthelmintic drugs demonstrated activity specifically against Gram-positive species from the ESKAPE pathogen panel (Fig 3 and Table 1). Obvious reasons for the difference in activity could be due to structural and molecular differences between the two classes of bacteria. The action of efflux pumps in transporting the drugs out of Gram-negative bacteria cannot also be ruled out. For example, efflux pumps have been implicated in the resistance of *P*. *aeruginosa* against tetracycline, chloramphenicol and norfloxacin [59, 60], agents that are quite effective against Gram-positive bacteria such as *S*. *aureus*. Moreover, the time-kill experiment data together with the MIC and MBC activity against MRSA suggest that niclosamide is bacteriostatic whereas oxyclozanide is bactericidal (Fig 4). Therefore even within susceptible bacteria, the drugs might have different modes of action.

In order to further investigate the mechanism of killing of MRSA by the anthelmintic drugs, their effect on the integrity of the bacterial cell envelope was examined. Oxyclozanide rapidly permeabilized the MRSA cell membrane after a short exposure as seen by the dose dependent increase in fluorescence caused by binding of Sytox Green dye with bacterial nucleic acid (Fig 5). No such observation was made for niclosamide. Although oxyclozanide targets the bacterial cell membrane, it does not cause disintegration of the bacterial cell since treatment of bacterial cells with these drugs resulted in no change in the bacterial density (data not shown). These findings suggest that further studies are needed to identify the exact mechanism of action of salicylanilide anthelmintic drugs against *S*. *aureus*. It is reasonable to assume that apart from targeting the bacterial cell envelope, oxyclozanide might also have other cellular targets which might also be the targets of niclosamide.

The activity of niclosamide and oxyclozanide against mammalian cell membranes was investigated by studying the hemolytic effect of these drugs on sheep erythrocytes. The positive control Triton X-100 displayed a dose dependent lysis of sheep RBCs, which was confirmed by both visual and spectrophotometric observation (Fig 6). In contrast, neither anthelmintic drug caused hemolysis. These findings are in agreement with earlier studies which indicate that it is not uncommon that compounds targeting bacterial cell membrane need not cause similar damage to mammalian cell membranes [42, 43]. The cytotoxicity of niclosamide and oxyclozanide were evaluated against HepG2 human liver carcinoma cells. Both drugs caused a dose dependent loss in cell viability but the toxicity was greater for niclosamide than oxyclozanide especially at lower concentrations (Fig 7). Similar results were obtained when the toxicity of niclosamide and oxyclozanide were evaluated against HEK293 human embryonic kidney cells (data not shown). It is not surprising that niclosamide appears to be toxic against HepG2 and HEK293 cells as similar observations have been made by other investigators [24, 61, 62]. Niclosamide was shown to block Wnt signaling in HEK293 cells [61]. The same study also reported that niclosamide inhibits Wnt signaling in prostate and breast cancer cells, directly inhibiting cancer cell proliferation and also promoting cancer cell apoptosis at concentrations as low as 0.4 μg/ml. Another study by Tinghong Ye et al. reported the potency of niclosamide against breast cancer both *in vitro* and *in vivo* [24]. Similar to these findings, Minoru Tomizawa et al. reported that niclosamide at a concentration of 0.3 μg/ml inhibited hepatic cancer cell proliferation through inhibition of the Wnt signaling pathway [62]. The findings from our study and the studies mentioned above suggest that niclosamide displays general toxicity to cancer cells and also blocks signaling pathways in other cell lines such as HEK293, which may lead to cytotoxicity. The results of the toxicity assay for oxyclozanide appear to be more interesting since the veterinary drug displayed lesser toxicity than niclosamide when tested on mammalian cells. Toxic effect of oxyclozanide was only seen starting at concentrations 16 fold higher than the MIC range against MRSA (Fig 7). These results do not necessarily indicate the relative safety of oxyclozanide versus niclosamide but suggests that it is worth initiating further studies to explore the possibility of repurposing oxyclozanide as a human drug. The results from this study suggest that there are both similarities and differences in the activities and toxicity profiles of niclosamide and oxyclozanide, not only between the two, but also with structural analogues reported in earlier studies. For example, Hilliard et al. [63] described a salicylanilide analogue that had good *in vitro* activity against *S*. *aureus* and was also capable of causing damage to both bacterial and erythrocyte membranes. However, the same study reported other salicylanilide analogues that were equally effective against *S*. *aureus* but did not have any effect on bacterial or erythrocyte membranes. The authors of another study [64] tested the effects of 32 salicylanilide derivatives on HepG2 cells and observed varying degrees of toxicities. The findings from our study and earlier studies suggest that niclosamide and oxyclozanide may have more than one mechanism. The relatively low toxicities of niclosamide and oxyclozanide that we observed is consistent with the fact that have been approved as human and veterinary therapeutics, respectively.

In conclusion, the salicylanilide anthelmintic drugs niclosamide and oxyclozanide inhibit growth of the Gram-positive bacteria *S*. *aureu*s and *E*. *faecium*. Moreover, since oxyclozanide targets the bacterial cell envelope, there is a low propensity of emergence of antimicrobial resistance against this drug. Further studies are needed to study the exact mode of action of these drugs and their pharmacokinetic and pharmacodynamics profiles. Since niclosamide is FDA approved and all of the salicylanilide anthelmintic drugs are already out of patent, they are attractive candidates for drug repurposing and warrant further clinical investigation for treating staphylococcal infections.

## Supporting Information

S1 Fig Chemical structures of the non-antimicrobial anthelmintic drugs from the Biomol 4 library. These anthelmintic drugs were present in the Biomol 4 library and they did not prolong survival of infected worms in the *C*. *elegans*-MRSA liquid infection assay.
(TIF)

S2 Fig Estimation of Minimal bactericidal concentration (MBC) of niclosamide and oxyclozanide. MBC against *E*. *faecium* **(A)** and *S*. *aureus* **(B)** was estimated by plating 10 μl of bacterial samples from the overnight MIC plates and observing bacterial growth on Müller-Hinton agar plates.
(TIF)

## References

[1] Boucher HW, Corey GR. Epidemiology of methicillin-resistant Staphylococcus aureus. Clinical infectious diseases: an official publication of the Infectious Diseases Society of America. 2008;46 Suppl 5:S344–9. 18462089 10.1086/533590

[2] Jones RN. Global epidemiology of antimicrobial resistance among community-acquired and nosocomial pathogens: a five-year summary from the SENTRY Antimicrobial Surveillance Program (1997–2001). Seminars in respiratory and critical care medicine. 2003;24(1):121–34. 16088531 10.1055/s-2003-37923

[3] Gottlieb GS, Fowler VG Jr., Kong LK, McClelland RS, Gopal AK, Marr KA, et al Staphylococcus aureus bacteremia in the surgical patient: a prospective analysis of 73 postoperative patients who developed Staphylococcus aureus bacteremia at a tertiary care facility. Journal of the American College of Surgeons. 2000;190(1):50–7. 10625232 10.1016/s1072-7515(99)00211-2

[4] Benfield T, Espersen F, Frimodt-Moller N, Jensen AG, Larsen AR, Pallesen LV, et al Increasing incidence but decreasing in-hospital mortality of adult Staphylococcus aureus bacteraemia between 1981 and 2000. Clinical microbiology and infection: the official publication of the European Society of Clinical Microbiology and Infectious Diseases. 2007;13(3):257–63. 10.1111/j.1469-0691.2006.01589.x 17391379

[5] Feng Y, Chen CJ, Su LH, Hu S, Yu J, Chiu CH. Evolution and pathogenesis of Staphylococcus aureus: lessons learned from genotyping and comparative genomics. FEMS microbiology reviews. 2008;32(1):23–37. 17983441 10.1111/j.1574-6976.2007.00086.x

[6] Lindsay JA. Genomic variation and evolution of Staphylococcus aureus. International journal of medical microbiology: IJMM. 2010;300(2–3):98–103. 10.1016/j.ijmm.2010.08.004 19811948

[7] Cuddy SM. Methicillin-resistant Staphylococcus aureus: a new pandemic? Plastic surgical nursing: official journal of the American Society of Plastic and Reconstructive Surgical Nurses. 2008;28(4):168–9. 19092579 10.1097/PSN.0b013e31818ea7ca

[8] (CDC) CfDCaP. Active Bacterial Core Surveillance (ABCs) Report Emerging Infections Program Network Methicillin-Resistant Staphylococcus aureus. 2011 [updated 2012]. Available: http://www.cdc.gov/abcs/reports-findings/survreports/mrsa11.pdf.

[9] Silver LL. Challenges of antibacterial discovery. Clinical microbiology reviews. 2011;24(1):71–109. 10.1128/CMR.00030-10 21233508 PMC3021209

[10] Schaberle TF, Hack IM. Overcoming the current deadlock in antibiotic research. Trends in microbiology. 2014;22(4):165–7. 10.1016/j.tim.2013.12.007 24698433

[11] Chopra S, Torres-Ortiz M, Hokama L, Madrid P, Tanga M, Mortelmans K, et al Repurposing FDA-approved drugs to combat drug-resistant Acinetobacter baumannii. The Journal of antimicrobial chemotherapy. 2010;65(12):2598–601. 10.1093/jac/dkq353 20861141

[12] Carlson-Banning KM, Chou A, Liu Z, Hamill RJ, Song Y, Zechiedrich L. Toward repurposing ciclopirox as an antibiotic against drug-resistant Acinetobacter baumannii, Escherichia coli, and Klebsiella pneumoniae. PloS one. 2013;8(7):e69646 10.1371/journal.pone.0069646 23936064 PMC3720592

[13] Weaver AJ Jr., Shepard JB, Wilkinson RA, Watkins RL, Walton SK, Radke AR, et al Antibacterial activity of THAM Trisphenylguanide against methicillin-resistant Staphylococcus aureus. PloS one. 2014;9(5):e97742 10.1371/journal.pone.0097742 24840307 PMC4026384

[14] Sifri CD, Begun J, Ausubel FM. The worm has turned—microbial virulence modeled in Caenorhabditis elegans. Trends in microbiology. 2005;13(3):119–27. 15737730 10.1016/j.tim.2005.01.003

[15] Mylonakis E, Casadevall A, Ausubel FM. Exploiting amoeboid and non-vertebrate animal model systems to study the virulence of human pathogenic fungi. PLoS pathogens. 2007;3(7):e101 17676994 10.1371/journal.ppat.0030101 PMC1933451

[16] Desalermos A, Tan X, Rajamuthiah R, Arvanitis M, Wang Y, Li D, et al. A Multi-Host Approach for the Systematic Analysis of Virulence Factors in Cryptococcus neoformans. The Journal of infectious diseases. 2014. 10.1093/infdis/jiu441 PMC4342695 25114160

[17] Rajamuthiah R, Mylonakis E. Effector triggered immunity: Activation of innate immunity in metazoans by bacterial effectors. Virulence. 2014;5(7). 10.4161/viru.29062 25513770 PMC4189875

[18] Sifri CD, Begun J, Ausubel FM, Calderwood SB. Caenorhabditis elegans as a model host for Staphylococcus aureus pathogenesis. Infection and immunity. 2003;71(4):2208–17. 12654843 10.1128/IAI.71.4.2208-2217.2003 PMC152095

[19] Irazoqui JE, Troemel ER, Feinbaum RL, Luhachack LG, Cezairliyan BO, Ausubel FM. Distinct pathogenesis and host responses during infection of C. elegans by P. aeruginosa and S. aureus. PLoS pathogens. 2010;6:e1000982 10.1371/journal.ppat.1000982 20617181 PMC2895663

[20] Ewbank JJ, Zugasti O. C. elegans: model host and tool for antimicrobial drug discovery. Disease models & mechanisms. 2011;4(3):300–4. 21504910 10.1242/dmm.006684 PMC3097103

[21] Rajamuthiah R, Fuchs BB, Jayamani E, Kim Y, Larkins-Ford J, Conery A, et al Whole Animal Automated Platform for Drug Discovery against Multi-Drug Resistant Staphylococcus aureus. PloS one. 2014;9(2):e89189 10.1371/journal.pone.0089189 24586584 PMC3929655

[22] Boneca IG, Chiosis G. Vancomycin resistance: occurrence, mechanisms and strategies to combat it. Expert opinion on therapeutic targets. 2003;7(3):311–28. 12783569 10.1517/14728222.7.3.311

[23] Macielag MJ, Demers JP, Fraga-Spano SA, Hlasta DJ, Johnson SG, Kanojia RM, et al Substituted salicylanilides as inhibitors of two-component regulatory systems in bacteria. Journal of medicinal chemistry. 1998;41(16):2939–45. 9685233 10.1021/jm9803572

[24] Ye T, Xiong Y, Yan Y, Xia Y, Song X, Liu L, et al The anthelmintic drug niclosamide induces apoptosis, impairs metastasis and reduces immunosuppressive cells in breast cancer model. PloS one. 2014;9(1):e85887 10.1371/journal.pone.0085887 24416452 PMC3885752

[25] Tao H, Zhang Y, Zeng X, Shulman GI, Jin S. Niclosamide ethanolamine-induced mild mitochondrial uncoupling improves diabetic symptoms in mice. Nature medicine. 2014;20(11):1263–9. 10.1038/nm.3699 25282357 PMC4299950

[26] Campbell WC. The chemotherapy of parasitic infections. The Journal of parasitology. 1986;72(1):45–61. 3519918

[27] The selection and use of essential medicines. Report of the WHO Expert Committee, 2002 (including the 12th Model list of essential medicines). 2003 0512–3054 (Print). 12872478

[28] Roy RM, Sukhla SS. Oxyclozanide-activity against Fasciola gigantica in naturally infected buffalo, cattle, sheep and goats. Tropical animal health and production. 1971;3(1):26–31. 5164589 10.1007/BF02356681

[29] Froyd G. Field trials with oxyclozanide. A new liverfluke remedy for sheep and cattle. The British veterinary journal. 1968;124(3):116–25. 5689150

[30] Cheng TJ, Wu YT, Yang ST, Lo KH, Chen SK, Chen YH, et al High-throughput identification of antibacterials against methicillin-resistant Staphylococcus aureus (MRSA) and the transglycosylase. Bioorganic & medicinal chemistry. 2010;18(24):8512–29. 21075637 10.1016/j.bmc.2010.10.036

[31] Kratky M, Vinsova J, Novotna E, Mandikova J, Trejtnar F, Stolakova J. Antibacterial activity of salicylanilide 4-(trifluoromethyl)-benzoates. Molecules. 2013;18(4):3674–88. 10.3390/molecules18043674 23529028 PMC6270420

[32] Pauk K, Zadrazilova I, Imramovsky A, Vinsova J, Pokorna M, Masarikova M, et al New derivatives of salicylamides: Preparation and antimicrobial activity against various bacterial species. Bioorganic & medicinal chemistry. 2013;21(21):6574–81. 24045008 10.1016/j.bmc.2013.08.029

[33] Kratky M, Bosze S, Baranyai Z, Szabo I, Stolarikova J, Paraskevopoulos G, et al Synthesis and in vitro biological evaluation of 2-(phenylcarbamoyl)phenyl 4-substituted benzoates. Bioorganic & medicinal chemistry. 2015;23(4):868–75. 25593095 10.1016/j.bmc.2014.12.019

[34] Tan MW, Mahajan-Miklos S, Ausubel FM. Killing of Caenorhabditis elegans by Pseudomonas aeruginosa used to model mammalian bacterial pathogenesis. Proceedings of the National Academy of Sciences of the United States of America. 1999;96(2):715–20. 9892699 10.1073/pnas.96.2.715 PMC15202

[35] Beanan MJ, Strome S. Characterization of a germ-line proliferation mutation in C. elegans. Development. 1992;116(3):755–66. 1289064 10.1242/dev.116.3.755

[36] Tanaka-Hino M, Sagasti A, Hisamoto N, Kawasaki M, Nakano S, Ninomiya-Tsuji J, et al SEK-1 MAPKK mediates Ca2+ signaling to determine neuronal asymmetric development in Caenorhabditis elegans. EMBO reports. 2002;3(1):56–62. 11751572 10.1093/embo-reports/kvf001 PMC1083920

[37] Jayamani E, Rajamuthiah R, Larkins-Ford J, Fuchs BB, Conery AL, Vilcinskas A, et al Insect-Derived Cecropins Display Activity against Acinetobacter baumannii in a Whole-Animal High-Throughput Caenorhabditis elegans Model. Antimicrobial agents and chemotherapy. 2015;59(3):1728–37. 10.1128/AAC.04198-14 25583713 PMC4325797

[38] Kamentsky L, Jones TR, Fraser A, Bray MA, Logan DJ, Madden KL, et al Improved structure, function and compatibility for CellProfiler: modular high-throughput image analysis software. Bioinformatics. 2011;27(8):1179–80. 10.1093/bioinformatics/btr095 21349861 PMC3072555

[39] Moy TI, Conery AL, Larkins-Ford J, Wu G, Mazitschek R, Casadei G, et al High-throughput screen for novel antimicrobials using a whole animal infection model. ACS chemical biology. 2009;4(7):527–33. 10.1021/cb900084v 19572548 PMC2745594

[40] Jorgensen JH, Ferraro MJ. Antimicrobial susceptibility testing: a review of general principles and contemporary practices. Clinical infectious diseases: an official publication of the Infectious Diseases Society of America. 2009;49(11):1749–55. 10.1086/647952 19857164

[41] Haste NM, Hughes CC, Tran DN, Fenical W, Jensen PR, Nizet V, et al Pharmacological properties of the marine natural product marinopyrrole A against methicillin-resistant Staphylococcus aureus. Antimicrobial agents and chemotherapy. 2011;55(7):3305–12. 10.1128/AAC.01211-10 21502631 PMC3122406

[42] Koh JJ, Qiu S, Zou H, Lakshminarayanan R, Li J, Zhou X, et al Rapid bactericidal action of alpha-mangostin against MRSA as an outcome of membrane targeting. Biochimica et biophysica acta. 2013;1828(2):834–44. 10.1016/j.bbamem.2012.09.004 22982495

[43] Isnansetyo A, Kamei Y. MC21-A, a bactericidal antibiotic produced by a new marine bacterium, Pseudoalteromonas phenolica sp. nov. O-BC30(T), against methicillin-resistant Staphylococcus aureus. Antimicrobial agents and chemotherapy. 2003;47(2):480–8. 12543647 10.1128/AAC.47.2.480-488.2003 PMC151744

[44] Rosch JW, Boyd AR, Hinojosa E, Pestina T, Hu Y, Persons DA, et al Statins protect against fulminant pneumococcal infection and cytolysin toxicity in a mouse model of sickle cell disease. The Journal of clinical investigation. 2010;120(2):627–35. 10.1172/JCI39843 20093777 PMC2810080

[45] Kwon B, Kumar P, Lee HK, Zeng L, Walsh K, Fu Q, et al Aberrant cell cycle reentry in human and experimental inclusion body myositis and polymyositis. Human molecular genetics. 2014;23(14):3681–94. 10.1093/hmg/ddu077 24556217 PMC4065145

[46] Van Den Bossche H, Verhoeven H, Vanparijs O, Lauwers H, Thienpont D. Closantel, a new antiparasitic hydrogen ionophore [proceedings]. Archives internationales de physiologie et de biochimie. 1979;87(4):851–3. 93944

[47] Rice LB. Progress and challenges in implementing the research on ESKAPE pathogens. Infection control and hospital epidemiology: the official journal of the Society of Hospital Epidemiologists of America. 2010;31 Suppl 1:S7–10. 10.1086/655995 20929376

[48] French GL. Bactericidal agents in the treatment of MRSA infections—the potential role of daptomycin. The Journal of antimicrobial chemotherapy. 2006;58(6):1107–17. 17040922 10.1093/jac/dkl393

[49] Martin RJ. Modes of action of anthelmintic drugs. Veterinary journal. 1997;154(1):11–34. 9265850 10.1016/s1090-0233(05)80005-x

[50] Swan GE. The pharmacology of halogenated salicylanilides and their anthelmintic use in animals. Journal of the South African Veterinary Association. 1999;70(2):61–70. 10855824 10.4102/jsava.v70i2.756

[51] Andrews P, Thyssen J, Lorke D. The biology and toxicology of molluscicides, Bayluscide. Pharmacology & therapeutics. 1982;19(2):245–95. 6763710 10.1016/0163-7258(82)90064-x

[52] Day SR, Moore CM, Kundzins JR, Sifri CD. Community-associated and healthcare-associated methicillin-resistant Staphylococcus aureus virulence toward Caenorhabditis elegans compared. Virulence. 2012;3(7):576–82. 10.4161/viru.22120 23076331 PMC3545934

[53] Begun J, Sifri CD, Goldman S, Calderwood SB, Ausubel FM. Staphylococcus aureus virulence factors identified by using a high-throughput Caenorhabditis elegans-killing model. Infection and immunity. 2005;73(2):872–7. 15664928 10.1128/IAI.73.2.872-877.2005 PMC547013

[54] Sifri CD, Baresch-Bernal A, Calderwood SB, von Eiff C. Virulence of Staphylococcus aureus small colony variants in the Caenorhabditis elegans infection model. Infection and immunity. 2006;74(2):1091–6. 16428756 10.1128/IAI.74.2.1091-1096.2006 PMC1360298

[55] Wu K, Simor AE, Vearncombe M, McClure JA, Zhang K. A Caenorhabditis elegans host model correlates with invasive disease caused by Staphylococcus aureus recovered during an outbreak in neonatal intensive care. The Canadian journal of infectious diseases & medical microbiology = Journal canadien des maladies infectieuses et de la microbiologie medicale / AMMI Canada. 2012;23(3):130–4. 10.1155/2012/543817 PMC3476557 23997780

[56] Kirienko NV, Kirienko DR, Larkins-Ford J, Wahlby C, Ruvkun G, Ausubel FM. Pseudomonas aeruginosa disrupts Caenorhabditis elegans iron homeostasis, causing a hypoxic response and death. Cell host & microbe. 2013;13(4):406–16. 23601103 10.1016/j.chom.2013.03.003 PMC3641844

[57] Desalermos A, Muhammed M, Glavis-Bloom J, Mylonakis E. Using C. elegans for antimicrobial drug discovery. Expert opinion on drug discovery. 2011;6(6):645–52. 21686092 10.1517/17460441.2011.573781 PMC3115622

[58] Hu Y, Ellis BL, Yiu YY, Miller MM, Urban JF, Shi LZ, et al An extensive comparison of the effect of anthelmintic classes on diverse nematodes. PloS one. 2013;8(7):e70702 10.1371/journal.pone.0070702 23869246 PMC3712009

[59] Li XZ, Livermore DM, Nikaido H. Role of efflux pump(s) in intrinsic resistance of Pseudomonas aeruginosa: resistance to tetracycline, chloramphenicol, and norfloxacin. Antimicrobial agents and chemotherapy. 1994;38(8):1732–41. 7986003 10.1128/aac.38.8.1732 PMC284630

[60] Li XZ, Ma D, Livermore DM, Nikaido H. Role of efflux pump(s) in intrinsic resistance of Pseudomonas aeruginosa: active efflux as a contributing factor to beta-lactam resistance. Antimicrobial agents and chemotherapy. 1994;38(8):1742–52. 7986004 10.1128/aac.38.8.1742 PMC284631

[61] Lu W, Lin C, Roberts MJ, Waud WR, Piazza GA, Li Y. Niclosamide suppresses cancer cell growth by inducing Wnt co-receptor LRP6 degradation and inhibiting the Wnt/beta-catenin pathway. PloS one. 2011;6(12):e29290 10.1371/journal.pone.0029290 22195040 PMC3241710

[62] Tomizawa M, Shinozaki F, Motoyoshi Y, Sugiyama T, Yamamoto S, Sueishi M, et al Niclosamide suppresses Hepatoma cell proliferation via the Wnt pathway. OncoTargets and therapy. 2013;6:1685–93. 10.2147/OTT.S50065 24273411 PMC3836661

[63] Hilliard JJ, Goldschmidt RM, Licata L, Baum EZ, Bush K. Multiple mechanisms of action for inhibitors of histidine protein kinases from bacterial two-component systems. Antimicrobial agents and chemotherapy. 1999;43(7):1693–9. 10390224 10.1128/aac.43.7.1693 PMC89345

[64] Zhu ZW, Shi L, Ruan XM, Yang Y, Li HQ, Xu SP, et al Synthesis and antiproliferative activities against Hep-G2 of salicylanide derivatives: potent inhibitors of the epidermal growth factor receptor (EGFR) tyrosine kinase. Journal of enzyme inhibition and medicinal chemistry. 2011;26(1):37–45. 10.3109/14756361003671060 20583855

[65] Garsin DA, Sifri CD, Mylonakis E, Qin X, Singh KV, Murray BE, et al A simple model host for identifying Gram-positive virulence factors. Proceedings of the National Academy of Sciences of the United States of America. 2001;98(19):10892–7. 11535834 10.1073/pnas.191378698 PMC58570

[66] Kuroda M, Ohta T, Uchiyama I, Baba T, Yuzawa H, Kobayashi I, et al Whole genome sequencing of meticillin-resistant Staphylococcus aureus. Lancet. 2001;357(9264):1225–40. 11418146 10.1016/s0140-6736(00)04403-2

[67] Rahme LG, Stevens EJ, Wolfort SF, Shao J, Tompkins RG, Ausubel FM. Common virulence factors for bacterial pathogenicity in plants and animals. Science. 1995;268(5219):1899–902. 7604262 10.1126/science.7604262

[68] Baba T, Bae T, Schneewind O, Takeuchi F, Hiramatsu K. Genome sequence of Staphylococcus aureus strain Newman and comparative analysis of staphylococcal genomes: polymorphism and evolution of two major pathogenicity islands. Journal of bacteriology. 2008;190(1):300–10. 17951380 10.1128/JB.01000-07 PMC2223734

[69] Nair D, Memmi G, Hernandez D, Bard J, Beaume M, Gill S, et al Whole-genome sequencing of Staphylococcus aureus strain RN4220, a key laboratory strain used in virulence research, identifies mutations that affect not only virulence factors but also the fitness of the strain. Journal of bacteriology. 2011;193(9):2332–5. 10.1128/JB.00027-11 21378186 PMC3133102

[70] Novick RP, Ross HF, Projan SJ, Kornblum J, Kreiswirth B, Moghazeh S. Synthesis of staphylococcal virulence factors is controlled by a regulatory RNA molecule. The EMBO journal. 1993;12(10):3967–75. 7691599 10.1002/j.1460-2075.1993.tb06074.x PMC413679

[71] McDougal LK, Steward CD, Killgore GE, Chaitram JM, McAllister SK, Tenover FC. Pulsed-field gel electrophoresis typing of oxacillin-resistant Staphylococcus aureus isolates from the United States: establishing a national database. Journal of clinical microbiology. 2003;41(11):5113–20. 14605147 10.1128/JCM.41.11.5113-5120.2003 PMC262524

[72] Mack D, Nedelmann M, Krokotsch A, Schwarzkopf A, Heesemann J, Laufs R. Characterization of transposon mutants of biofilm-producing Staphylococcus epidermidis impaired in the accumulative phase of biofilm production: genetic identification of a hexosamine-containing polysaccharide intercellular adhesin. Infection and immunity. 1994;62(8):3244–53. 8039894 10.1128/iai.62.8.3244-3253.1994 PMC302952

