# Supplementary figures and images for: Repurposing Salicylanilide Anthelmintic Drugs to Combat Drug Resistant Staphylococcus aureus
Source: PLoS One. 2015 Apr 21;10(4):e0124595. doi: 10.1371/journal.pone.0124595 (PMC4405337; doi:10.1371/journal.pone.0124595)

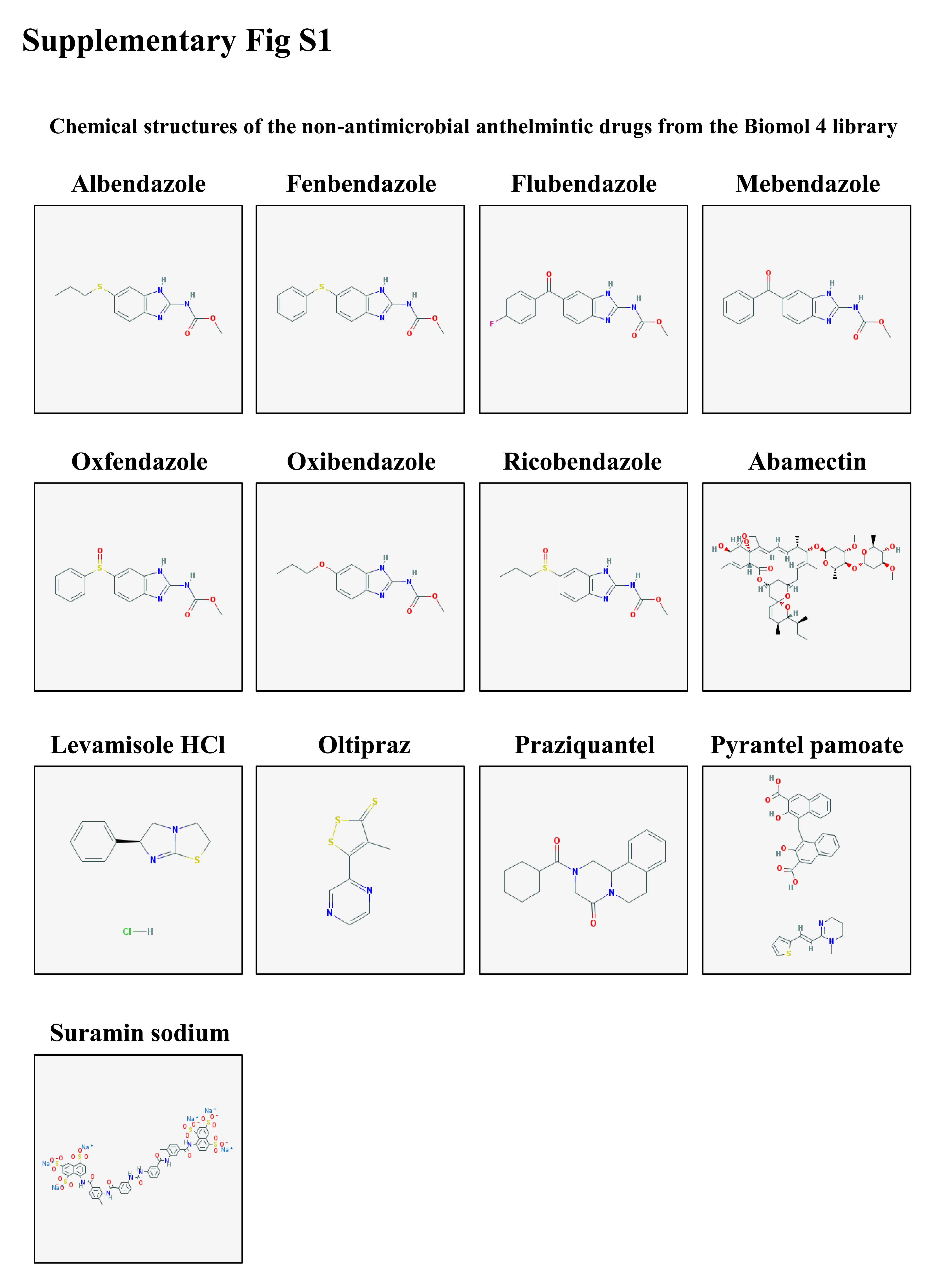

Supplement: S1 Fig — These anthelmintic drugs were present in the Biomol 4 library and they did not prolong survival of infected worms in the C. elegans-MRSA liquid infection assay. (TIF) [file pone.0124595.s001.tif]

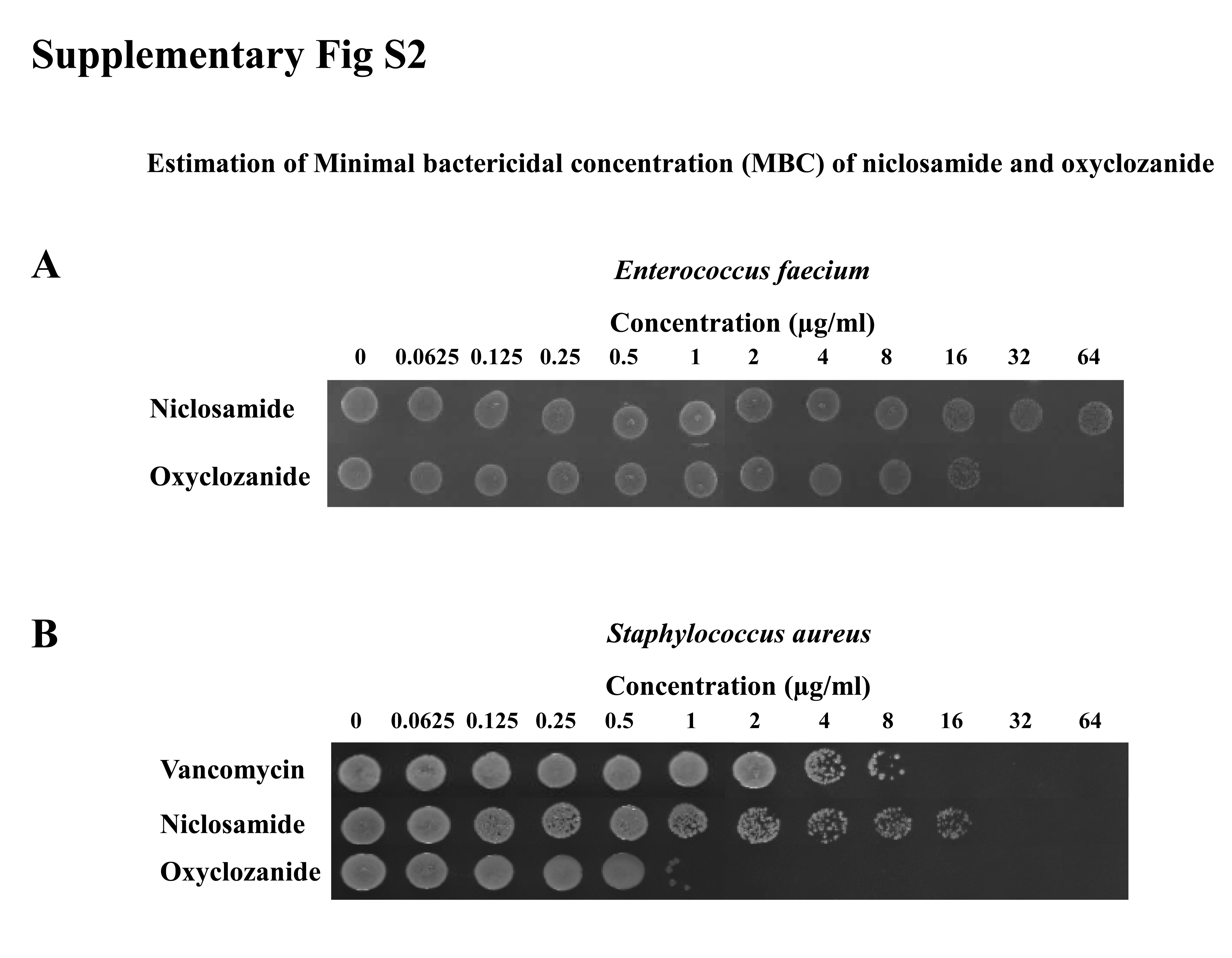

Supplement: S2 Fig — MBC against E. faecium (A) and S. aureus (B) was estimated by plating 10 μl of bacterial samples from the overnight MIC plates and observing bacterial growth on Müller-Hinton agar plates. (TIF) [file pone.0124595.s002.tif]
